# Multiple facial and left eye injuries in a 13 day old baby secondary to rat bite

**DOI:** 10.11604/pamj.2014.17.311.3964

**Published:** 2014-04-24

**Authors:** Waheed Ademola Ibraheem, Anifat Boladale Ibraheem, Ajagbe Kayode Ibraheem

**Affiliations:** 1 Department of Ophthalmology, Ladoke Akintola University Teaching Hospital, Ogbomoso, Oyo State, Nigeria; 2 Department of Family Medicine, University College Hospital, Ibadan, Oyo State, Nigeria

**Keywords:** Facial injury, rat bite, microbial keratitis

## Abstract

A case of traumatic blepharectomy secondary to a rat bite in a 13 day old neonate. Infants should not be kept in an isolated place in a rat endemic area. This case suggests an existence of a relationship between poverty and rat bite.

## Introduction

Although, ocular injuries from animal bite are not uncommon, those caused by rat are relatively scarce or maybe poorly reported. However, when rat eye injury occurred, it is relatively commoner in the infants and more often than not the incidence usually happens when the victims are asleep [1]. In a study conducted in Baltimore from 1939 through 1943, 60% of the victims were < 1 year of age and most of the bites occurred in an area of substandard housing [1]. Other idenfiable risk factors are; poverty, unemployment and rat infestations [2].

## Patient and observation

We report a case of multiple facial injuries of a 13 day old neonate who was well until 90mins prior to presentation when mother's attention was drawn to the patient by the older sibling who noticed she was crying. She noticed blood on the left side of the face of the child and on cleaning observed complete loss of the left upper and lower eyelids, partial loss of the nares and an ulcer on the left cheek. No eye witness account of the exact cause of the incident. About 30 minutes prior to the incident, she was breastfed, wrapped in a shawl and put in a zipped up net that was placed on a bed about 60cm from the floor. She was alone in the room after breastfeeding while her mother was attending to other domestic needs in the backyards.

Patient is a second set of a preterm twin and the fourth children of the second wife in a polygamous family setting. Father is a primary school dropout and he is a lorry driver. Mother is a secondary school dropout and she is a sewing mistress by profession. The patient lives with her other siblings in a single room in a rented apartment which has no fence. Mother also reported free mobility and navigation of rats within and around the house before the incidence. No similar incidence in the other siblings.

Patient is a second set of a preterm twin and the last of the four children of the second wife in a polygamous family setting. Father is a primary school dropout and he is a lorry driver. Mother is a secondary school dropout and she is a sewing mistress by profession. The patient lives with her other siblings in a single room in a rented apartment which has no fence. Mother also reported free mobility and navigation of rats within and around the house before the incidence. No similar incidence in the other siblings.

**Figure 1:**
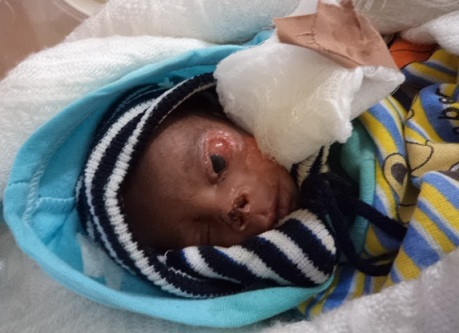
Picture showing multiply injured neonate involving left upper and lower lids, tip of nose and left cheek

Examination revealed an acutely ill looking child with complete loss of the left upper and lower eyelids, partial loss of both nares, 2 by 3cm ulcer on the left cheek and left central corneal ulcer (Figure 1). Her weight was1kg, occipitofrontal circumference was, left arm circumference was 37cm and cranial caudal length was 22cm. She was neither pale nor febrile. The right eye with and the adnexae were intact. Systemic examinations were not contributory. She was admitted into special baby care unit (SCBU) of the hospital and was reviewed by the pediatrician who recommended that she should be in the incubator, and was given subcutaneous ante tetanus toxoid 750 IU stat after negative test dose, intramuscular TT 0.5ml stat, intravenous (IV) Unasyn 150mg/kg/dy in 3 divided doses, IV Gentamicin 5mg/kg/day. She was also fed with EBM at 120kcal/kg/day. The wound was cleaned daily with normal saline after review by the plastic surgeon. Generous chloramphenicol ointment was applied over the ocular injury while the globe was protected by Catellar shield during her sleep and at night. She was also commenced on artifial eye tear hourly.

**Figure 2:**
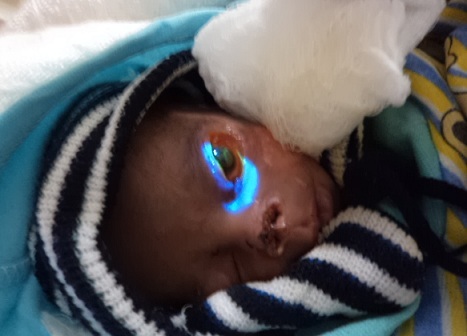
Picture showing left microbial keratitis deeply stained with 2% fluorescein dye

**Figure 3:**
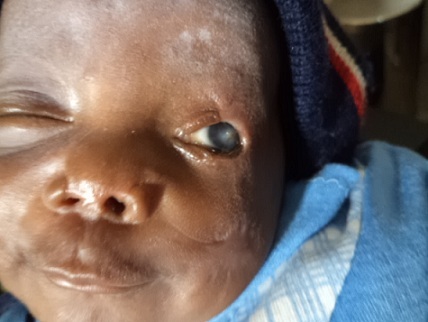
Picture showing residual left cornea opacity and deformed left eyelids

Two weeks after the injury she was noticed to have developed left central microbial keratitis (Figure 2). Examination at this time revealed a deeply stained central corneal ulcer with circum-corneal injection. The care giver was offered a referral to a corneal specialist at an outside facility but they declined due to financial constraint. Patient was to have corneal scrapings done for microscopic, culture and sensitivity but the care giver could also not afford it. She was commenced on hourly Lomefloxacillin (3%) eye drop which was procured through voluntary donations from the physicians. The corneal ulcer and infiltrates have resolved (Figure 3) and the care giver has been informed of the need for penetrating keratoplasty and lid reconstruction as soon as possible. The child is currently doing well under routine follow-up at ophthalmology, pediatrics and plastic surgery departments.

## Discussion

The management of rat bite injury includes the immediate first aid and definitive medical or surgical treatment depending on the severity and the extent of the injury. It is desirable that the wound should be rinsed thoroughly with clean water like tap water, debrided and covered with a sterile dressing [3]. Tap water has been shown to be as effective for irrigation as sterile saline. Treatment recommendations include wound debridement and avoidance of prophylactic antibiotics due to a low natural infection rate. Where there is risk of exposure keratopathy such as in case of severe lid loss, temporary measures like the use of bandage contact lens, Catellar shield could be employed while the patient awaits definitive lid reconstruction surgery. Rabies prophylaxis is not routinely advocated but tetanus prophylaxis is mandatory in rat bite injury [4].

Ordog and co-investigators conducted a prospective study on 50 patients with rat bite to determine the natural incidence of wound infection without prophylactic antibiotics [4]. The authors reported infection in only 2% of their study population (I patient) and concluded that routine treatment for rat bite should include good surgical management and avoidance of prophylactic antibiotics because of the low natural infection rate in rat bite [4].

Contrarily, Marshall and co-researcher reported a case of recurrent multiple abscess in a 24 old woman whose pets was rat. She became free from recurrent abscess only when she received courses of antibiotics and was denied access to rat [5]. This report signify the role of prophylactic antibiotics in the management of rat bite injury.

Generally, there is no one specific lid reconstruction procedure for blepharectomy due to rat injury. The choice of appropriate procedure depends on the extent of the tissue loss and the lid that is involved. For a large lower lid loss, the Mustarde cheek flap technique (MCFT) is useful. It is a large, rotational, skin-muscle cheek flap that be relied on to cover virtually any lower lid defect [6]. The advantage of this procedure is that it is a one-stage, complete lower lid reconstruction. A graft of either hard palate or ear cartilage is needed for posterior lamellar support. For a large upper lid loss accompanied with lower lid tissue loss, a large, horizontal advancement of an upper eyelid tarsoconjunctival flap is useful. The anterior lamella can be replaced with a full-thickness skin graft.

## Conclusion

Further awareness should be created on the occurrence of rodent eye injury and prevention programs should be directed to high-risk populations.
